# Mycological Survey and Antifungal Susceptibility Evaluation of *Candida albicans* Isolates in European Hedgehogs (*Erinaceus europaeus*)

**DOI:** 10.3390/vetsci12040306

**Published:** 2025-03-28

**Authors:** Leonardo Brustenga, Giulia Morganti, Marco Gobbi, Alice Ranucci, Giulia Rigamonti, Iolanda Moretta, Manuela Diaferia, Nicoletta D’Avino, Deborah Cruciani, Marcella Ciullo, Francesca Romana Massacci, Silvia Crotti

**Affiliations:** 1 Department of Veterinary Medicine, University of Perugia, Via San Costanzo 4, 06126 Perugia, Italy; 2 WildUmbria Wildlife Rescue Center, 06026 Pietralunga, Italy; 3 Istituto Zooprofilattico Sperimentale dell’Umbria e delle Marche “Togo Rosati”, Via Gaetano Salvemini 1, 06126 Perugia, Italy

**Keywords:** European hedgehogs, dermatophytes, yeasts, *Paraphyton mirabile*, *Candida albicans*

## Abstract

European hedgehogs are an important reservoir for many pathogens of health interest. Since hedgehogs live in close contact with humans, potential zoonotic fungi raise significant public health concerns, especially in areas with a high hedgehog density. From 2020 to 2023, 134 hedgehogs were surveyed for potential zoonotic fungi. Non-invasive methods were used, such as brushing live animals with a sterile toothbrush and taking oral and rectal swabs from deceased ones (86 animals). Dermatophytes were cultured on Dermasel agar and identified using molecular tools, while yeasts were isolated on Sabouraud agar with chloramphenicol and determined using Candida Chromogenic agar (MicroBiolDiagnostici^®^, Cagliari, Italy) and MALDI-TOF (Microflex LT Smart Biotyper with FlexControlBiotyper 3.4 software, Bruker Daltonics, Bremen, Germany). Minimum inhibitory concentrations (MICs) were determined for *Candida albicans* isolates. Dermatophytes were found in just one hedgehog (0.8%, 95% C.I.: 0–0.04), identified as *Paraphyton mirabile*. Yeasts were detected in 22 of 86 hedgehogs (25.6%, 95% C.I.: 16.4–34.8), with 25 isolates obtained, including 21 *Candida albicans*, 2 *Yarrowia lipolytica*, 1 *Rhodotorula mucilaginosa*, and 1 *Meyerozyma guilliermondii*. All *C. albicans* isolates showed a high susceptibility to the antimycotic panel tested. Monitoring zoonotic fungi harbored by European hedgehogs, as well as raising public awareness on the topic, is of great importance for public health.

## Simple Summary

European hedgehogs can pose health risks to humans, particularly in areas where they live in close contact. A study of 134 hedgehogs (2020–2023) examined potential zoonotic fungi. While dermatophytes were rare (with only one case of *Paraphyton mirabile* observed), yeasts were more common, detected in 25.6% of the sampled hedgehogs. The most frequent yeast was *Candida albicans*, followed by *Yarrowia lipolytica*, *Rhodotorula mucilaginosa*, and *Meyerozyma guilliermondii*. *Candida albicans* isolates showed high susceptibility to antifungal treatments. This study highlights the importance of monitoring fungal species in wildlife and raising public awareness to protect human health.

## 1. Introduction

The European hedgehog (*Erinaceus europaeus* Linnaeus, 1758) is a small mammal widely distributed across Western and Central Europe [1]. Many studies have shown a substantial decline in the European hedgehog population in Northern Europe (the UK, Belgium, the Netherlands, Sweden, and Germany), likely caused by habitat loss and fragmentation, molluscicides and rodenticide poisoning, intensive agricultural practices, road traffic, and predation from European badgers (*Meles meles* Linnaeus, 1758) in some areas [2]. According to two consecutive assessments conducted by International Union for the Conservation of Nature (IUCN 2013, 2022), there is no evidence of a decline in the Italian population of European hedgehogs, which remains abundant and is not particularly affected by human factors such as traffic [3].

Hedgehogs have nocturnal habits and an omnivorous diet, mostly feeding on arthropods and small invertebrates, such as earthworms and gastropods, that are dug up from the soil or actively hunted in the leaf litter [1,4]. Many of these invertebrates act as intermediate or paratenic hosts for several parasites such as bronchopulmonary nematodes *Crenosoma striatum* and *Eucoleus aerophilus*, and the fluke *Brachylaemus erinacei*, which can cause severe and sometimes fatal infestations in hedgehogs [4,5,6]. Furthermore, many of these parasites have a zoonotic potential, including *Cryptosporidium* spp. [7] and *Giardia duodenalis* [8], or can be transmitted to both humans and pets, such as *E. aerophilus* [5,6]. Moreover, hedgehogs can also harbor several harmful and potentially zoonotic bacteria from the genera *Borrelia*, *Coxiella*, *Leptospira*, *Klebsiella*, and *Salmonella* [9], with a high percentage of antimicrobial resistance [10]. Lastly, European hedgehogs constitute an important reservoir for various health-related fungi that inhabit their integument [11,12], such as *Trichophyton erinacei*, a zoophilic dermatophyte that was first isolated in hedgehogs from New Zealand, raising relevant public health concerns [13,14,15]. Hedgehogs are usually asymptomatic carriers, lacking detectable clinical lesions, whereas direct and indirect transmission can cause dermatophytosis on both humans [16,17,18] and dogs [19,20]. Mite infestations, sustained by *Caparinia tripilis*, which can cause scabbing and furfural shedding [9], have been shown to facilitate the transmission of zoonotic dermatophytes, such as *Microsporum canis* and *Microsporum gypseum* [21], or *Trichophyton erinacei* [22]. Moreover, hedgehogs can serve as reservoirs for geophilic fungi that are of low zoonotic concern [11]. Among the mycotic pathogens harbored by hedgehogs, infections caused by the commensal yeast *Candida albicans* have also been reported [9]. There is, in fact, evidence of both oral and intestinal candidiasis in European hedgehogs (*E. europaeus*) and African pygmy hedgehogs (*Atelerix albiventris* Wagner, 1841) [23,24].

Considering the numerous pathogens harbored by hedgehogs, it is important not to overlook the health of urban populations, as this may pose a threat to both humans and domestic animals. Since hedgehogs tend to live in close proximity to humans by foraging or hibernating in gardens and public parks, improper handling without the use of adequate protections, or practices that attract hedgehogs to human spaces—such as providing food and water sources shared with domestic animals—can facilitate the transmission of zoonotic pathogens from the wild to the domestic environment.

A mycological survey on pathogenic fungi was carried out to investigate dermatophytes and yeasts on European hedgehogs rescued from Central Italy. Furthermore, minimum inhibitory concentrations (MICs) were tested to interpret the susceptibility and resistance levels of the detected mycotic pathogens to the available antifungal drugs.

## 2. Materials and Methods

A total of 134 wild European hedgehogs rescued by WildUmbria Wildlife Rescue Center (Central Italy) between 2020 and 2023 were admitted to the University of Perugia Veterinary Teaching Hospital for emergency care and enrolled in the survey. Of these, 48 of the 134 animals were alive at the time of sampling, while 86 out of 134 were deceased due to causes independent of this study (i.e., road traffic or predation victims) and were sampled during necroscopic procedures. Sample collection for dermatophyte investigations was carried out via the toothbrush technique [25]. Samples from live animals were taken on admission by gently brushing the integument of the exposed parts without forcing the animal to uncurl to avoid stressful procedures; samples from deceased animals were taken by brushing all the available surfaces. The samples were inoculated on Dermasel agar, incubated at 25 ± 1 °C, and observed daily for 14 days. Oral and rectal swabs were also collected from deceased animals during the necroscopic inspections to detect yeasts. These samples were sown on Sabouraud Dextrose agar (SDA) supplemented with chloramphenicol and Candida Chromogenic agar (MicroBiolDiagnostici^®^, Cagliari, Italy) and incubated at 37 °C for 24–48 h. The identification of fungal growths attributable to dermatophytes and yeasts was based on visual inspection of macroscopic and microscopic features with the support of the mycology identification keys provided by The University of Adelaide (https://www.adelaide.edu.au/mycology/ accessed on 12 December 2024); accurate species identification was achieved through molecular and mass spectrometry investigations.

Dermatophyte colonies grown on Dermasel agar were subjected to DNA extraction using the QIAamp DNA mini kit (QIAGEN^®^, Hilden, Germany) following a modified Gram-positive protocol (Appendix C of the handbook: Protocols for Bacteria. https://www.qiagen.com/us/resources/resourcedetail?id=62a200d6-faf4-469b-b50f-2b59cf738962&lang=en, accessed on 12 December 2024). An end-point PCR was carried out to confirm dermatophyte isolation using universal fungal primers ITS1 (5′-TCCGTAGGTGAACCTGCGG-3′) and ITS4 (5′-TCCTCCGCTTATTGATATGC-3′) [26]. PCR amplification was carried out at a total volume of 50 µL. The reaction mixture was prepared as follows: 5X Colorless GoTaq^®^ Flexi Buffer (Promega, Madison, WI, USA), 2.5 mM of MgCl_2_ (Promega, Madison, WI, USA); 0.2 mM of each dNTP (Global Life Sciences Solutions Operations, Little Chalfont, UK); 0.4 µM of each primer; 1.25 units of GoTaq^®^ Hot Start Polymerase (Promega, Madison, WI, USA); 3 µL of template DNA and Nuclease-Free Water (Thermo Fisher Scientific, Austin, TX, USA). PCR amplification was performed in a Mastercycler Nexus X2 (Eppendorf AG, Hamburg, Germany) set to the following conditions: denaturation at 95 °C for 5 min; 35 cycles of 94 °C for 45 s, 56 °C for 45 s, and 72 °C for 1 min; and a final extension at 72 °C for 10 min. The PCR product was run in a 2% agarose gel containing Midori Green Advance (NIPPON Genetics^®^, Europe GmbH, Düren, Germany). Gel electrophoresis on a 2% agarose gel stained with Midori Green Advance DNA stain (NIPPON Genetics Europe GmbH^®^, Düren, Germany) allowed for the visualization of the PCR products. The amplicons were purified using a QIAquick PCR Purification Kit (QIAGEN^®^, Hilden, Germany) to perform a sequencing reaction using a BrilliantDye^TM^ Terminator v3.1 Cycle Sequencing Kit (NimaGen^®^, Nijmegen, The Netherlands). The obtained forward and reverse sequences were run in a 3500 Genetic Analyzer (Applied Biosystem, Foster City, CA, USA). Eventually, the consensus sequences were created by BioEdit Sequence Alignment Editor software v7.2.5 [27] and aligned in the Westerdijk Fungal BioDiversity Institute database.

Yeast colonies grown on SDA and Candida Chromogenic agar were identified through a Matrix-Assisted Laser Desorption/Ionization Time-Of-Flight (MALDI-TOF) instrument (Microflex LT Smart Biotyper with FlexControlBiotyper 3.4 software, Bruker Daltonics, Bremen, Germany). MALDI-TOF MS analysis was performed directly from the subcultures obtained on agar plates. A small quantity of the yeast colony was smeared on a spot of a 96-spot stainless steel target plate (Bruker Daltonics, Bremen, Germany). Then, 1 μL of a 70% formic acid solution overlapped each spot. After that, 1 μL of HCCA matrix (a-cyano-4-hydroxycinnamic acid supplied with MALDI-TOF reagents by Bruker Daltonics, Bremen, Germany) was applied on each spot and air-dried before analysis with MALDI-TOFMS. The Bruker Biotyper 3.4 software and library were used for spectral analysis. Following the manufacturer’s instructions, scores ≥ 2.0 were interpreted as successful identifications at the genus and species levels. Furthermore, *C. albicans* strains were also tested for the sensitivity to the most common antifungals using the broth micro-dilution method utilizing Sensititre^TM^ YeastOne YO10^®^ (Thermo Fisher Scientific, Waltham, MA, USA) according to the manufacturer’s instructions. Colonies grown on SDA and Candida Chromogenic agar were suspended in sterile water, adjusted to the 2 McFarland standard set (Liofilchem Dianostici ^®^, Roseto degli Abruzzi, Italy), and diluted in the yeast broth; a 100 µL of the obtained suspension was dispensed in each well. The plates were incubated at 37 °C for 24–48 h. The concentration in the first blue-colored well was recorded as the minimum inhibitory concentration (MIC). The antifungal susceptibility of specific *Candida* species was interpreted through clinical breakpoints (CBPs) based on the CLSI M60 [28,29]. The antifungal molecules tested included anidulafungin, micafungin, caspofungin, 5-fluorocytosine, posaconazole, voriconazole, itraconazole, fluconazole, and amphotericin B.

## 3. Results

Clinical examinations did not reveal skin lesions such as spike loss, crusty skin, or erythema, which could suggest dermatophytosis; similarly, deceased animals sampling during the necroscopic inspections to detect yeasts did not exhibit mucosal lesions suggestive of mycosis.

**Figure 1:**
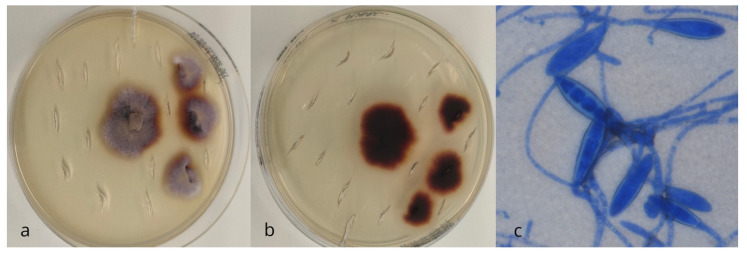
Recto (**a**) and versus (**b**) macroscopic morphology and microscopic (methylene blue stain, 40×) (**c**) features of *Paraphyton* spp. colonies.

Dermatophytes were identified in just 1 of the 134 hedgehogs (0.8%, 95% C.I.: 0–0.04). The colonies that grew on Dermasel agar appeared as flat, ramified, powdery-like, and pale brown (Figure 1a). The reverse pigment appeared to be dark reddish-brown with some radial grooves (Figure 1b). Numerous large, very thick-walled, elliptical macroconidia with predominantly five to six septa were observed microscopically (Figure 1c). The strain was genetically identified as *Paraphyton mirabile* (GenBank accession number PV242270).

**Figure 2:**
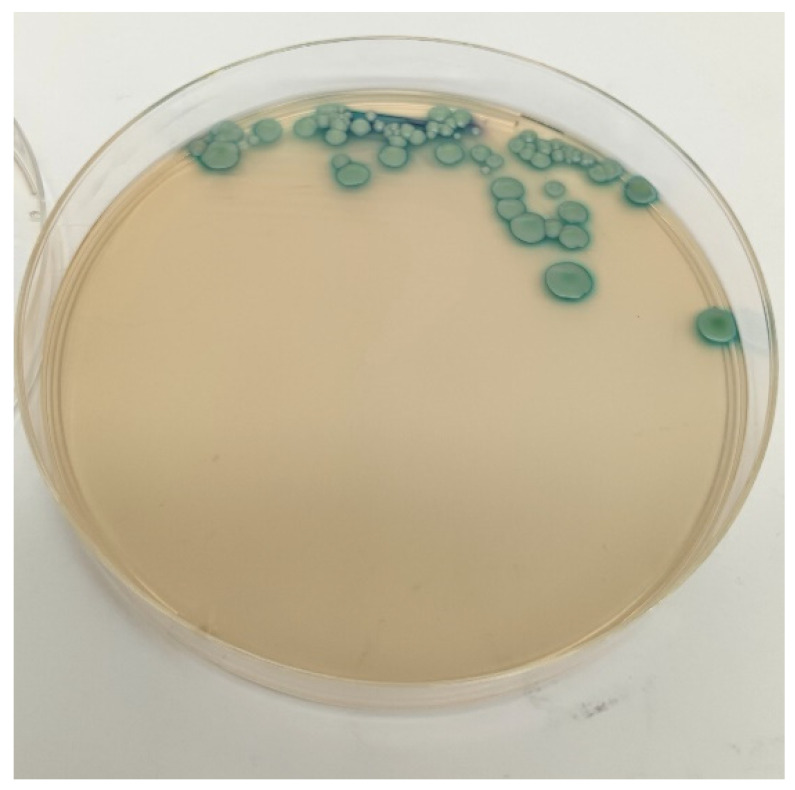
*Candida albicans* colonies on *Candida* Chromogenic agar.

Yeasts were detected in 22 of the 86 deceased hedgehogs (25.6%, 95% C.I.: 16.4–34.8), and a total of 25 different strains were isolated, as yeasts were detected in both oral and rectal swabs of 3 animals. Twenty-one strains were isolated both on Sabouraud Dextrose agar and on Candida Chromogenic agar, appearing as light green colonies (Figure 2) and resembling the chromatic phenotype of the *C. albicans* reference strain.

MALDI-TOF confirmed the identification of *C. albicans* from the chromogenic agar, as well as the identification of other strains, such as two *Yarrowia lipolitica*, one *Rhodotorula mucilaginosa*, and one *Meyerozyma guilliermondii* (the anamorph name of *Candida guillermondii*) [30] (Table 1).

Moreover, the 21 *C. albicans* strains were susceptible to the whole panel of the antimycotics tested, with MIC values tending to fall within the lower end of the reported sensitivity range (Table 2). Among azoles, voriconazole (MICm 0.01; sensitivity range: 0.008–1), posaconazole (MICm 0.03; sensitivity range: 0.008–1) and fluconazole (MICm 0.3; sensitivity range: 0.12–8) exhibited lower MIC values, while itraconazole showed more variability among isolates, with a slightly high MIC value (MICm 0.05; sensitivity range: 0.015–0.12). Echinocandins, such as anidulafungin (MICm 0.02; sensitivity range: 0.015–2) and micafungin (MICm 0.01; sensitivity range: 0.008–2), were effective on the isolates at the lower range value tested. Amphotericin B demonstrated relatively consistent MIC values across isolates (MICm 0.26; sensitivity range: 0.12–1).

## 4. Discussion

Hedgehog populations have increased in urban areas, becoming synanthropic animals and expanding their opportunity for interactions with other animals and humans. This is evidenced by reports on infectious zoonotic diseases associated with hedgehogs, including their ability to transmit dermatophytes to humans [9]. Dermatophytosis caused by the zoonotic *Trichophyton erinacei* has been frequently documented in both free-ranging and captive hedgehogs, with prevalence rates as high as 25 to 50% [11,12,31,32,33]. Although to a lesser extent, geophilic dermatophytes such as *Nannizzia gypsea*, *P. cookei* and *P. mirabile* have also been reported, constituting minor zoonotic concerns, generally in asymptomatic animals [11,34]. Thus, the detection of dermatophytes in just one hedgehog was rather unexpected. However, considering that the reservoir for geophilic dermatophytes is the soil, their detection highlights the need for greater attention to soil contamination caused by wildlife, as it can indirectly pose a risk of infection to humans and other animals, including pets [11,34].

In this survey, parasitic yeasts were also detected. Some of them, such as *Rhodotorula mucilaginosa* and *Meyerozyma guillermondii*, are emerging opportunistic pathogens that can cause mycotic infections in both humans and animals [35,36]. An interesting finding is the isolation and the identification of *C. albicans*, which was previously identified as a commensal organism in the digestive tracts of hedgehogs [9]. Finding *C. albicans* in oral swabs suggests the potential for transmission through bites or contamination of the hedgehog’s integument due to self-anointing behavior [37]. Since *C. albicans* is the causative agent of candidiasis in humans and animals, its detection in the oral mucosa or integument suggests that these animals should be considered potential carriers or reservoirs for this yeast. This highlights the importance of implementing proper management practices, particularly in immunosuppressed individuals (e.g., elderly people and infants), who may accidentally handle these synanthropic animals. Despite the potential hazard, the MIC values for *C. albicans* obtained in this study are encouraging, showing that all isolates are susceptible to the antifungal drugs that were tested. Regular and more extensive fungal monitoring is encouraged to confirm this trend. Nevertheless, it must be considered that pathogenic fungi possess numerous resistance mechanisms to antifungal drugs, facilitated by their genetic adaptability and versatile homeostatic responses to environmental stressors. Currently, the available literature data highlight the decreased efficacy of certain antifungal drugs against some dermatophytes and yeasts of the genus *Malassezia* in domestic animals [14,38,39]. Wildlife has recently been recognized as a significant bioindicator not only of environmental quality, but also as a sentinel for many zoonotic pathogens, as well as for assessing the presence of antimicrobial resistance genes contamination in the environment [40,41,42,43,44]. Further research on larger samples and, possibly, more species of wild animals should be carried out to assess the diffusion of fungal diseases and their resistance to antifungal treatments. Monitoring the presence of antifungal resistance is particularly important, especially in animals that are rescued and rehabilitated for reintroduction into the wild, as this practice can promote the spreading of antifungal resistant strains, posing a risk to people who work in wildlife rehabilitation centers and come into daily contact with potentially infected animals.

These data highlight the critical importance of passive surveillance efforts in detecting zoonotic fungi in wild animals, thereby increasing awareness of the complex interactions between wildlife, public health, and the environment, following the One Health approach [44,45]. Considering wild animals as sentinels, investigating their health status is essential to prevent potential outbreaks of zoonotic infections and monitor the drug resistance phenomenon, which is an issue of increasing concern in both human and veterinary medicine. Effective strategies, such as discouraging wildlife habituation to human presence and implementing measures to reduce accessible food sources, are crucial in regions where the urban and natural environments overlap. Additionally, attention should be paid to those who closely interact with wildlife, not only wildlife rehabilitators, veterinarians, and technicians, but also and especially ordinary citizens who might come in contact with wild animals inhabiting gardens or public parks. Dedicated educational initiatives should then be promoted to highlight the health risks associated with improper wildlife handling.

## Figures and Tables

**Table 1:** Fungal isolates obtained through different sampling methods.

	*Paraphyton mirabile* Positives/ Total Samples (%)	*Candida albicans* Positives/ Total Samples (%)	*Yarrowia lipolytica* Positives/ Total Samples (%)	*Rhodotorula mucilaginosa* Positives/ Total Samples (%)	*Meyerozyma guilliermondii* Positives/ Total Samples (%)
Toothbrush	1/134 (0.8%)	0/134	0/134	0/134	0/134
Oral swab	Na	12/86 * (14.0%)	0/86	1/86 (1.16%)	1/86 (1.16%)
Rectal swab	Na	9/86 * (10.47%)	2/86 (2.33%)	0/86	0/86
N° of isolates	1	21	2	1	1

* *Candida albicans* isolates were from 18 animals, 3 of which were positive both for oral and rectal swabs; Na: Not applicable.

**Table 2:** Minimum inhibitory concentration (MIC, µg/mL) data of anidulafungin (NDF), micafungin (MCF), caspofungin (CSP), 5-flucytosine (FCT), posaconazole (POS), voriconazole (VOR), itraconazole (ITR), fluconazole (FLU), amphotericin (AMB) for *Candida albicans* (n. 21 isolates) from European hedgehogs.

***Candida albicans*** **(n. Isolates = 21)**	**Isolates**	**NDF**	**MCF**	**CSP**	**FCT**	**POS**	**VOR**	**ITR**	**FLU**	**AMB**
	Sensitivity RANGE	0.015–2	0.008–2	0.008–2	0.06–4	0.008–1	0.008–1	0.015–0.12	0.12–8	0.12–1
MIC	Isolate 01	0.015	0.015	0.03	0.12	0.03	0.015	0.03	0.25	0.25
	Isolate 02	0.015	0.015	0.06	0.12	0.015	0.015	0.03	0.25	0.25
	Isolate 03	0.015	0.015	0.06	0.12	0.015	0.015	0.03	0.25	0.25
	Isolate 04	0.015	0.015	0.06	0.12	0.015	0.015	0.03	0.5	0.25
	Isolate 05	0.015	0.03	0.03	0.25	0.015	0.008	0.03	0.25	0.25
	Isolate 06	0.03	0.015	0.03	0.25	0.015	0.015	0.03	0.25	0.25
	Isolate 07	0.015	0.008	0.03	0.06	0.03	0.015	0.06	0.25	0.25
	Isolate 08	0.015	0.008	0.06	0.06	0.03	0.008	0.06	0.5	0.25
	Isolate 09	0.015	0.015	0.06	0.06	0.015	0.008	0.12	0.25	0.25
	Isolate 10	0.015	0.015	0.06	0.06	0.03	0.015	0.06	0.25	0.25
	Isolate 11	0.015	0.015	0.06	0.06	0.03	0.015	0.06	0.25	0.25
	Isolate 12	0.015	0.015	0.03	0.12	0.03	0.015	0.06	0.25	0.25
	Isolate 13	0.015	0.015	0.03	0.12	0.03	0.015	0.06	0.25	0.25
	Isolate 14	0.015	0.015	0.03	0.06	0.03	0.015	0.06	0.25	0.25
	Isolate 15	0.015	0.015	0.03	0.12	0.03	0.015	0.06	0.5	0.25
	Isolate 16	0.015	0.015	0.015	0.12	0.03	0.015	0.06	0.5	0.25
	Isolate 17	0.03	0.008	0.03	0.06	0.03	0.008	0.03	0.25	0.25
	Isolate 18	0.015	0.015	0.06	0.06	0.03	0.008	0.03	0.25	0.25
	Isolate 19	0.015	0.015	0.06	0.06	0.03	0.008	0.03	0.25	0.25
	Isolate 20	0.015	0.015	0.06	0.06	0.03	0.008	0.03	0.25	0.5
	Isolate 21	0.015	0.015	0.03	0.06	0.03	0.015	0.06	0.25	0.25
MICm		0.02	0.01	0.04	0.10	0.03	0.01	0.05	0.30	0.26

MICm: MIC medium value.

## Data Availability

The data that support the findings of this study are available from the corresponding author, Giulia Morganti, upon reasonable request.
